# Mitochondrial dysfunction signatures in idiopathic primary male infertility: a validated proteomics-based diagnostic approach

**DOI:** 10.3389/frph.2024.1479568

**Published:** 2024-12-12

**Authors:** Raneen Sawaid Kaiyal, Sromona D. Mukherjee, Manesh Kumar Panner Selvam, Aaron W. Miller, Sarah C. Vij, Scott D. Lundy

**Affiliations:** ^1^ Glickman Urological Institute, Cleveland Clinic Foundation, Cleveland, OH, United States; ^2^ Department of Cardiovascular and Metabolic Sciences, Cleveland Clinic Foundation, Cleveland, OH, United States; ^3^ Department of Urology, Tulane University School of Medicine, New Orleans, LA, United States

**Keywords:** sperm proteome, spermatozoa protein, idiopathic male infertility, mitochondrion, reactive oxygen species, bioinformatics

## Abstract

**Research question:**

Male infertility accounts for almost half of all infertility cases worldwide, with idiopathic male infertility accounting for up to 30% of the cases. Sperm proteomics has revealed critical molecular pathway changes in men with infertility. However, the sperm mitochondrial proteome remains poorly understood. We attempted to answer the following question: Do patients with idiopathic primary male infertility exhibit a proteomic signature associated with mitochondrial dysfunction that could be used as a target for future mechanistic investigations?

**Design:**

Patients with idiopathic primary infertility (20–40 years old) referred to the Cleveland Clinic between March 2012 and April 2014 were compared with fertile donor controls. Sperm proteins were analyzed using sodium dodecyl sulphate-polyacrylamide gel electrophoresis page (SDS-PAGE) and liquid chromatography-mass spectrometry (LC-MS), and differentially expressed proteins (DEPs) were identified based on significance test results and fold change thresholds. Protein expression was validated using western blotting.

**Results:**

Proteomic analysis of pooled samples from fertile donors (*n* = 5) and patients with idiopathic primary infertility (*n* = 5) identified 1,134 proteins, including 344 DEPs. Mitochondrial dysfunction topped the ingenuity toxicity list. Analysis of expression levels of three mitochondrial proteins known to combat oxidative stress revealed that peroxiredoxin-5 (PRDX5) and superoxide dismutase 2 (SOD2), but not glutathione disulphide reductase, were significantly decreased in patient samples compared with those in fertile-donor samples.

**Conclusions:**

This study revealed an association of downregulated expression of PRDX5 and SOD2 in sperm samples of patients with idiopathic primary male infertility. Our results support future mechanistic studies and development of advanced diagnostic methods to better identify men with mitochondria-related male infertility.

## Introduction

Infertility is a widespread global health problem affecting an average of 17.5% of couples worldwide (1). Approximately 50% of these cases are attributed to male-factor infertility, either as the primary reason or as a significant contributing factor (2). The causes of male infertility vary and can be broadly classified as congenital, acquired, and idiopathic, each of which can negatively affect the crucial process of spermatogenesis (3). An idiopathic etiology accounts for 30% of all reported cases (4). Potential complex causes include factors such as defective sperm DNA integrity (5) abnormal sperm transcripts (6), and disrupted sperm protein expression, (7) all of which impair critical processes such as fertilization, the acrosome reaction, sperm-oocyte fusion, and embryo growth and development (8).

Spermatozoa, which are transcriptionally and translationally quiescent, depend entirely on existing proteins to perform essential biological functions (9). To date, research on the sperm proteome has identified and characterized different numbers of proteins (7, 10, 11) and the latest published number on sperm proteome is 7,000 gene products (12). These proteins play essential roles in energy production, apoptosis, oxidative stress response, cytoskeleton structure, flagellar movement, cell recognition, protein transport, and mitochondrial function (13, 14).

Mitochondria are the most important organelles in spermatozoa, providing cellular energy through ATP production to facilitate viability (15). Each spermatozoon contains between 50 and 75 mitochondria, which are located in the mid-piece and exhibit unique characteristics (16). Mitochondria are involved in several key functions, such as calcium homeostasis, generation of reactive oxygen species (ROS) (17–19), regulation of apoptotic pathways, and biosynthesis of steroid hormones (20). Defective mitochondrial function in spermatozoa has been linked to various pathologies and activities, including varicocele (21) and diabetes, leading to disorders in spermatogenesis due to the overproduction of ROS and inhibition of cell proliferation (22–24). Furthermore, mitochondrial damage has been linked to repetitive fertilization failure after intracytoplasmic sperm injection (ICSI) (25) and cigarette smoking in males (26). Studies have highlighted the importance of proper mitochondrial function for male fertility and reproductive health (15, 27, 28).

Some proteomics studies have analyzed the proteome of asthenozoospermia patients vs. normozoospermic individuals, with a predominant focus on mitochondrial proteins. Yet, there has been no clear clarification regarding whether the patients had idiopathic etiology or if the control group were fertile or infertile normozoospermic samples (29, 30). A recent study by Pacheco et al., analyzed the proteome of male infertility of unknown origin, including both idiopathic and unexplained male infertility samples (31). However, their control group included male partners from infertile couples who were normozoospermic with a female factor infertility. These individuals may not represent truly fertile normozoospermic males, as male factor infertility could also contribute to the infertility in these couples.

To date, there are no studies yet that have evaluated the proteome of idiopathic primary male infertility compared to proven fertile donors and using liquid chromatography-mass spectrometry (LC-MS) to evaluate any association between idiopathic primary male infertility and mitochondrial function. Consequently, no diagnostic tests based on the detection of mitochondrial proteins, are available to clinically diagnose mitochondrial dysfunction and idiopathic primary male infertility.

In this pilot study, we investigated whether patients with idiopathic primary male infertility exhibit a proteomic signature in the sperm that could potentially be used to identify mitochondrial dysfunction and treat the condition.

## Materials and methods

### Ethics statement

This study was conducted at the Andrology Centre, Cleveland Clinic, Cleveland, OH, USA. The proteomics data utilized were derived from our proteomic project on male infertility (IRB#11-451) in which we conducted multiple proteomic analyses on infertility patients with various etiologies, such as varicocele, primary infertility, and secondary infertility. In this study, we chose to focus on a group of idiopathic primary infertility patients. Our hypothesis evolved from preliminary findings in our broader proteomic analysis. For Western blot validation, we recruited a different set of participants following approval by the Cleveland Clinic Institutional Review Board (IRB#17-422). All individuals who participated in this study provided informed consent after signing a consent form.

We followed the recommendations for biomarker identification and qualification in clinical proteomics, the EQUATOR Network (32).

### Sample and data collection

Patients (*n* = 5) with idiopathic primary infertility between the ages of 20–40 years old, who were referred to the Glickman Urological and Kidney Institute at Cleveland Clinic were included in this study. Idiopathic primary infertility was diagnosed under the following criteria: (1) Semen analysis showed at least one abnormality regarding concentration, motility, or normal sperm morphology, in line with the fifth edition of the World Health Organization (WHO) 2010 guidelines (33), (2) No specific cause for infertility or semen pathology was identified, (3) A normal fertility assessment was confirmed for the female partner. The control group (*n* = 5) consisted of fertile donor participants who were in good health, exhibited a normal body mass index, were non-smokers, refrained from alcohol consumption, displayed normozoospermia, with no high oxidative stress defined as ROS levels <93 RLU/sec (Relative Light Units per second), and had successfully fathered a child within the preceding two years. The exclusion criteria for both groups included smoking, female factor infertility, exposure to radiation or chemicals, fever in the previous 90 days, genetic defects (such as Y-chromosome microdeletions and karyotype abnormalities), leukocytospermia, infection or inflammation of the reproductive tract, sexually transmitted diseases, and azoospermia.

To ensure adequate protein concentration for subsequent proteomic analysis, a sample pooling strategy was adopted for both the patient and donor groups. A protein concentration-normalization procedure was implemented for each pooled sample to achieve diverse amalgamations of the samples within each group. This procedure ensured an equal contribution of protein from an equivalent number of spermatozoa per sample within the pool. This was achieved using an aliquot of 75 μl from samples exhibiting a protein concentration of 1.5 mg/ml, derived from a sperm concentration ranging between 80 and 100 million spermatozoa/ml.

For a comprehensive global proteomic analysis, each sample from both the control (fertile donor) and patient groups underwent triplicate runs and analysis. This approach ensured the robustness and reliability of the obtained results.

### Semen analysis

Semen samples were collected at the Andrology Laboratory through masturbation, following a period of sexual abstinence lasting at least 2–7 days. The samples were allowed to liquefy completely for 30 min at 37°C, and semen analysis was conducted in accordance with the WHO (2010) (33) guidelines using a disposable Leja sperm counting chamber (Spectrum Technologies, Healdsburg, CA) to assess sperm count, motility, and morphology. After the routine semen analysis, samples were centrifuged at 13,000 g for 20 min. The seminal plasma was then removed, and the sperm samples were stored at −80°C.

### Protein extraction and characterization

**Figure 1:**
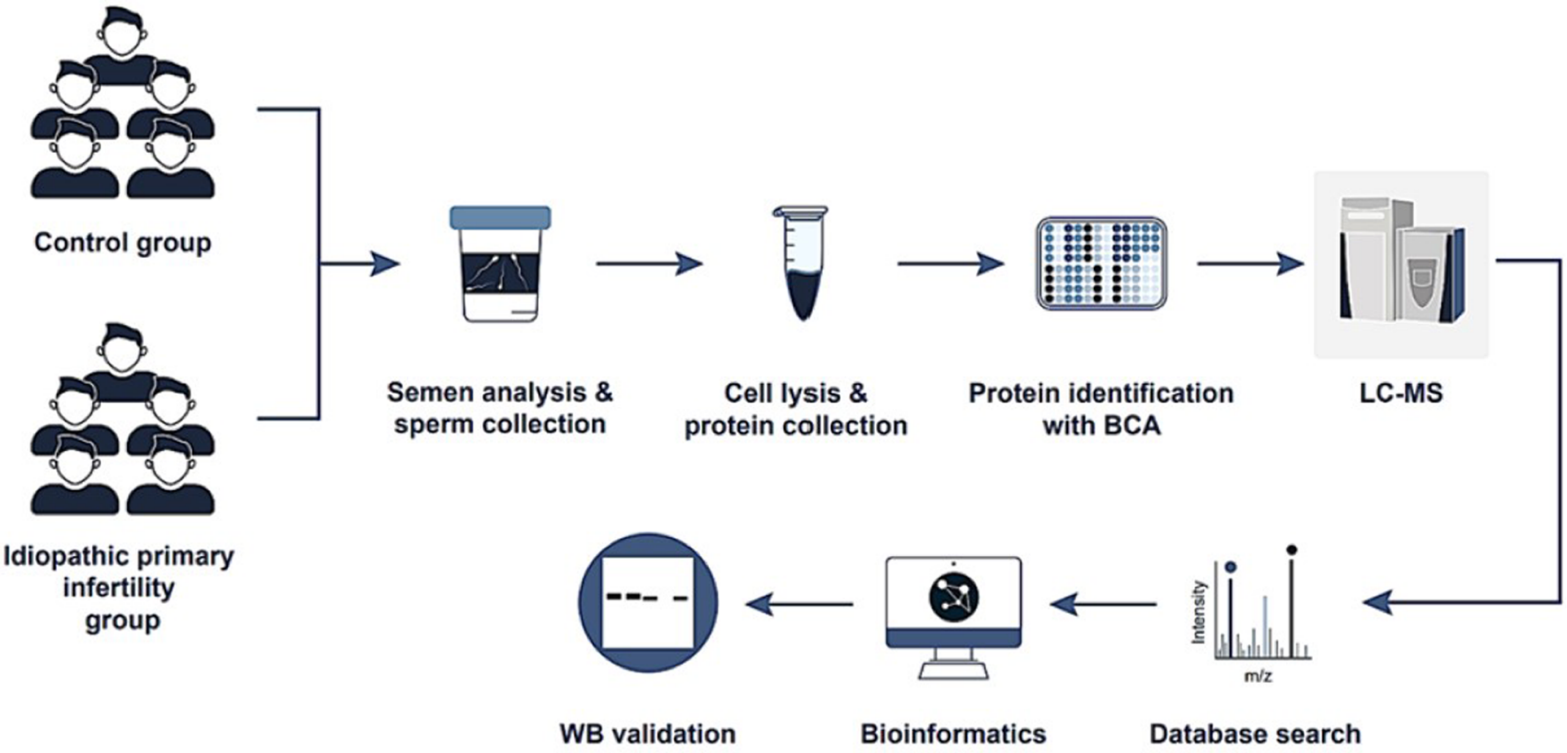
Study flowchart.

A detailed description of our protein-related research methods, including extraction, proteomics analysis, liquid chromatography-mass spectrometry (LC-MS) analysis, database searching, criteria for identification, and quantitative proteomics, is provided in Supplementary Material. In addition, a study flowchart is provided in Figure 1. The analysis was conducted in strict compliance with the Minimum Information about a Proteomics Experiment guidelines established by the Human Proteome Organization's Proteomics Standards Initiative (HUPO-PSI), a framework detailed in a 2013 publication by (34).

### Bioinformatics analysis

We primarily utilized Ingenuity Pathway Analysis (IPA) from Ingenuity® Systems to perform functional pathway analysis of the differentially expressed proteins (DEPs). IPA facilitated the examination of the top canonical pathways, diseases, biofunctions, causal networks, and upstream regulators associated with the DEPs. In addition, we employed publicly available bioinformatics annotation tools and databases, including GO Term Finder (35), STRING database (36), UniProt for researching annotations of proteins, and the Database for Annotation, Visualization and Integrated Discovery (DAVID) (37). These resources enriched our analysis and allowed a comprehensive exploration of protein annotations and associated information.

### Protein selection and validation using western blotting

To validate the results of our bioinformatics analysis, we employed western blot techniques using different sample sets from patients with idiopathic primary infertility and healthy donors. We successfully recruited four individuals for each group, adhering to the established inclusion and exclusion criteria previously used for proteomic analysis recruitment.

An aliquot of protein (20 µg) for each sample was resolved in a 4%–15% acrylamide gel using SDS-PAGE for 2 h at 90 V. Subsequently, the proteins were transferred onto polyvinylidene difluoride (PVDF) membranes and blocked for 90 min at room temperature of 20°C–25°C using a 5% non-fat milk solution in tris-buffered saline tween-20. For protein analysis, the membranes were incubated with the following specific primary antibodies overnight at 4°C: peroxiredoxin-5 (PRDX5; P30044, ab180587; Abcam, Cambridge, UK), superoxide dismutase 2 (SOD2; P04179, ab137037; Abcam), and glutathione disulphide reductase (GSR; P00390, ab128933; Abcam). Following incubation, the membranes were incubated with secondary Anti-Rabbit IgG VHH Single Domain (HRP; Abcam) at room temperature of 20°C–25°C for 1 h and then exposed to enhanced chemiluminescence (ECL) reagent (GE Healthcare, Marlborough, MA, USA) for 5 min. Chemiluminescence signals were detected using a Chemi-Doc™ MP Imaging System (Bio-Rad, Hercules, CA, USA). Glyceraldehyde 3-phosphate dehydrogenase (GAPDH) was used as a loading control, and the expression of each protein was normalized against GAPDH expression. We used HeLa Cells (ab157396; Abcam) as positive controls.

All the PVDF membranes used for protein identification were also subjected to total protein staining. The membranes were washed twice for 10 min in distilled water before incubating with total colloidal gold protein (Bio-Rad) for 2 h at room temperature with gentle shaking. After staining, the membranes were washed twice with distilled water for 10 min each, and densitometry images were captured in colorimetric mode using the Chemi-Doc™ MP Imaging System (Bio-Rad).

### Statistical analysis

To assess the semen parameters, we used a combination of statistical tests. Analysis of variance (ANOVA) and two-sample *t*-tests were used to compare the quantitative measurements of protein expression between the infertile patients and fertile donors. For the statistical comparison of proteomic data, individual *t*-tests were performed to compare pairs of groups. Specifically, for each protein, we conducted a two-sample *t*-test to assess differences in expression between two groups (infertile patients vs. fertile controls). While no adjustments were made for multiple comparisons, this approach allowed for a focused examination of the proteins of interest in our study. The analysis was carried out using MedCalc Statistical Software (version 17.8; MedCalc Software, Ostend, Belgium), with statistical significance considered at *p* < 0.05.

To compare the results of the semen analysis we employed the Mann-Whitney test, with significance established at *P* < 0.05. Additionally, a two-tailed Student's *t*-test was used to compare the western blot intensity readings.

## Results

### Semen analysis

The parameters of the semen samples, including the concentration, motility and morphology are shown in Table 1. Fertile donors exhibited normal sperm parameters while idiopathic infertile patients presented with at least one abnormal sperm parameter. While all patients had high level of abnormal sperm morphology, no signiﬁcant difference was observed in the sperm concentration, motility, and morphology between the two groups.

**Table 1:** Semen parameters.

	Fertile healthy donors (*n* = 5)	Idiopathic infertility patients (*n* = 5)	*P*-value
Sperm concentration (10^6^/ml)	61.6 (114.2, 52.5)^a^	19.4 (117.6, 15.5)	0.47
Sperm motility (%)	56 (58, 49.5)	58 (78, 21.5)	0.83
Normal sperm morphology (%)	7 (4, 13)	2 (1, 3)	0.06

^a^ Results are presented as median (25th, 75th percentile); *P* < 0.05 indicates a significant difference based on the Mann-Whitney test.

### Proteomic profiling

Proteomic analysis of pooled seminal samples from the fertile donor cohort (*n* = 5) and patients with idiopathic primary infertility (*n* = 5) led to the identification of 1,134 proteins overall, with a subset of 141 proteins was present exclusively in the fertile donor group, nine proteins were present exclusively in patients with idiopathic primary infertility and 344 proteins were deferentially expressed (as described in the Supplementary Material A) with 235 proteins underexpressed and 109 proteins overexpressed. The 1,134 proteins fell into distinct abundance categories: 559 were categorized as very low abundance, 273 as low abundance, 224 as medium abundance, and 78 as high abundance (see description in Supplementary Material B).

### Bioinformatics analysis

**Figure 2:**
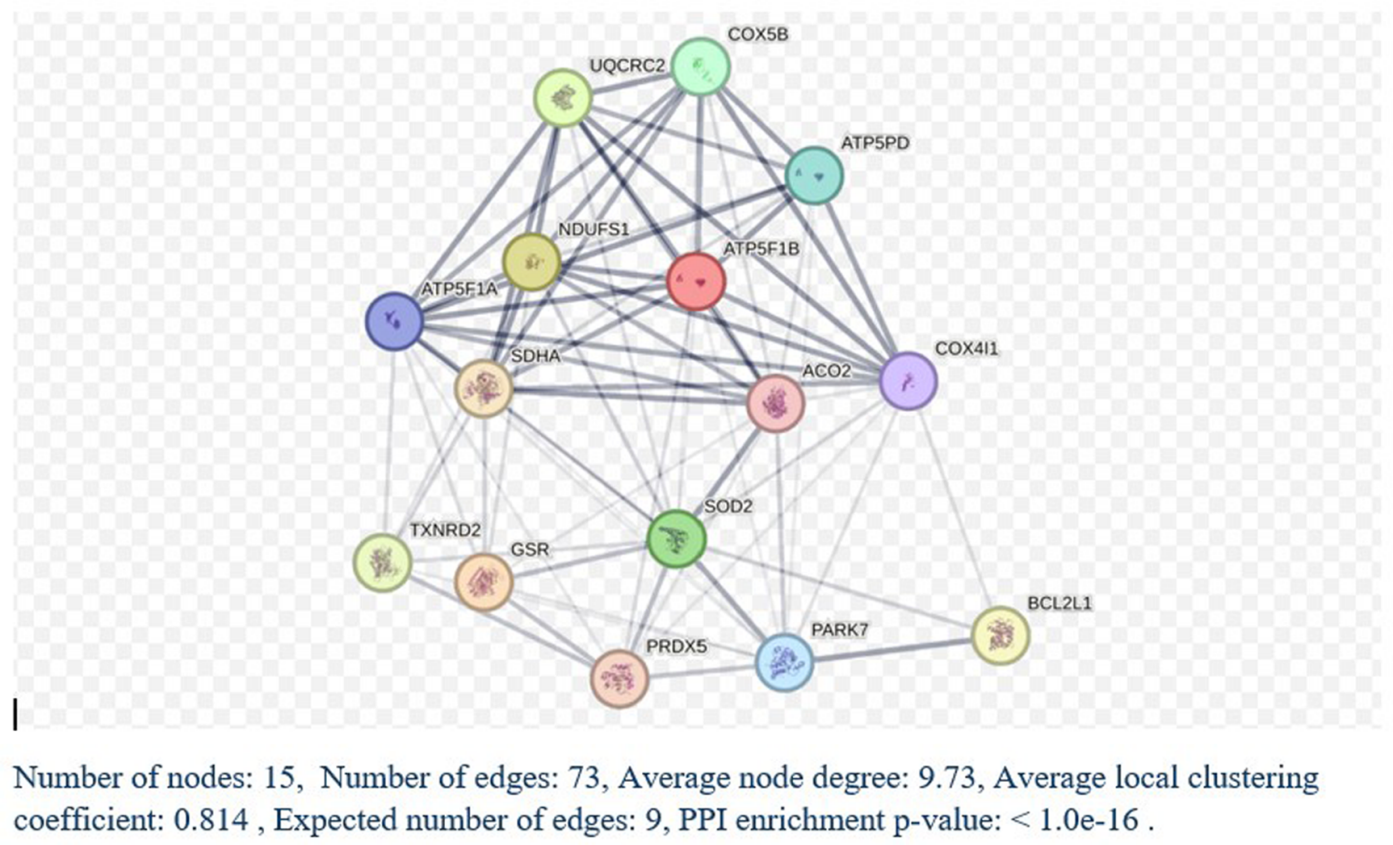
STRING analysis of protein-protein interactions. Network Status: number of nodes, 141; number of edges, 711; average node degree, 10.1; expected number of edges, 290; average local clustering coefficient, 0.433, and protein-protein interaction (PPI) enrichment *P*-value < 1.0E-16. ^a^ Count in network refers to the number of DEPs included in the STRING interaction network.

We utilized DAVID software for functional annotation analysis to elucidate the roles of the proteins exclusively expressed in fertile donors, proteins exclusively expressed in patients with idiopathic infertility and for DEPs in terms of biological processes, cellular components, and molecular function. The proteins that were unique to fertile donors were involved in electron transport, aerobic respiration, electron transport, ubiquitin system, cilium movement and protein folding. Proteins unique to patients with idiopathic infertility were involved in regulation od DNA replication and transcription, post translation modification, cell cycle G1/S phase and cell myosis (Table 2). Seventy-nine clusters were reported for functional annotation clustering, and 78 DEPs were involved in mitochondrial function, thirty four in mitochondrial matrix and 28 in mitochondrial inner membrane (Table 3). In addition, we harnessed the capabilities of IPA software to conduct a more comprehensive investigation into the associated canonical pathways, diseases, and biofunctions of the identified DEPs. Tables 4, 5 summarize the top disease and biofunction results. Mitochondrial dysfunction was at the top of the tox list with 8.8% overlap (15/171), (*P* = 1.78E–11). DEPs were involved in skeletal and muscular disorders, organismal injury and abnormalities, endocrine system disorder, neurological disease, and hereditary disorder. Most of the DEP's of them were involved in cell death and survival (*n* = 103), small molecule biochemistry (*n* = 51), nucleic acid metabolism (*n* = 29), translation modification (*n* = 18) and protein folding (*n* = 18). In addition, we also noticed that 32 DEP were involved in reproductive system development and function*.* Fifteen proteins from our DEPs were associated with the mitochondrial dysfunction pathways [-log (*p*-value) = 10.7 and ratio = 0.0877]: ACO2, ATP5F1A, ATP5F1B, ATP5PD, COX4I1, COX5B, GSR, NDUFS1, PARK7, PRDX5, SDHA, SOD2, TXNRD2, UQCRC2, and VDAC. We uploaded these 15 proteins to the STRING database for predicted protein-protein interaction networks and enrichment analysis, which revealed 73 interaction networks, as summarized in (Figure 2).

**Table 2:** Functional annotation clustering using DAVID bioinformatics resources for the unique proteins identified in fertile donors and patients with idiopathic infertility.

Fertile donors	Electron transport, Aerobic respiration, Electron transport, NADH to ubiquinone, Ubiquitin-dependent protein catabolic process, Cilium movement, Protein folding.
Patients with idiopathic infertility	Regulation of DNA replication, Post-translational protein modification, Mitotic cell cycle phase transition, Cell cycle G1/S phase transition, Positive regulation of transcription, cell division, Cell cycle, Cell, Mitosis.

**Table 3:** Functional annotation clustering using DAVID bioinformatics resources for differentially expressed proteins.

Key function	Number of proteins	*P*-value
Mitochondrion	78	2.60E-22
Transit peptide	45	6.60E-21
Mitochondrial matrix	34	2.40E-15
Mitochondrial inner membrane	28	9.30E-09

**Table 4:** Top tox list generated using IPA software.

Name	*P*-value	Overlap^a^
Mitochondrial dysfunction	1.78E-11	8.8% 15/171
Fatty acid metabolism	6.82E-06	6.8% 8/117
Oxidative stress	8.38E-06	10.5% 6/57
Renal necrosis/cell death	1.28E-04	2.5% 16/648

^a^ Overlap refers to the number of shared proteins identified from the DEP's set and different databases.

**Table 5:** Top diseases and biofunctions generated using IPA software.

Main diseases and disorders	Number of molecules	*P*-value range
Endocrine system disorder	169	1.71E-05–2.90E-16
Organismal injury and abnormalities	196	1.71E-05–2.90E-16
Hereditary disorder	70	1.15E-05–1.05E-14
Neurological disease	158	1.20E-05–1.05E-14
Skeletal and muscular disorders	690	9.87E-06–1.05E-14
Molecular and cellular functions	Number of molecules	*P*-value range
Post-translational modification	18	5.81E-06–6.04E-19
Protein folding	18	5.81E-06–6.04E-19
Nucleic acid metabolism	29	8.20E-06–7.03E-16
Small molecule biochemistry	51	8.20E-06–7.03E-16
Cell death and survival	103	1.52E-06–1.69E-12
Physiological system development	Number of molecules	*P*-value range
Reproductive system development and function	32	1.32E-05–1.32E-09
Cardiovascular system development and function	25	9.87E-06–5.72E-06
Organ morphology	21	1.20E-05–5.72E-06
Organismal development	41	1.23E-05–5.72E-06
Renal and urological system development and function	13	1.23E-05–5.72E-06

From the pool of identified proteins, we selected GSR, SOD2, and PRDX5 for subsequent western blot validation. The selection of these proteins was driven by several important factors relevant to our study. First, these proteins exhibited significant expression changes in our bioinformatic analysis of infertile patients (Table 6), making them highly pertinent to our research. Furthermore, they play essential roles in the male reproductive system and have well-documented involvement in mitochondrial function, which aligns with our central hypothesis of mitochondrial dysfunction in idiopathic infertility. Although these proteins were not the top DEPs, we prioritized them due to their strong association with mitochondrial processes, a key focus of our investigation. Additionally, the availability of reliable antibodies and positive controls allowed for robust validation via western blot. In our proteomics analysis, SOD2 and PRDX5 were underexpressed, while GSR was overexpressed, underscoring their potential significance in the pathophysiology of male infertility (Supplementary Material B).

**Table 6:** Expression fold change and *P*-values for the validated proteins.

Symbol	Entrez gene name	GI number	Expression fold change	*P*-value	Location
PRDX5	Peroxiredoxin 5	6,912,238	−19.253	0.208	Cytoplasm
SOD2	Superoxide dismutase 2	67,782,305	−47.245	0.029	Cytoplasm
GSR	Glutathione disulphide reductase	50,301,238	−93.206	0.017	Cytoplasm

GI number, GenBank identifier.

### Western blotting validation

**Figure 3:**
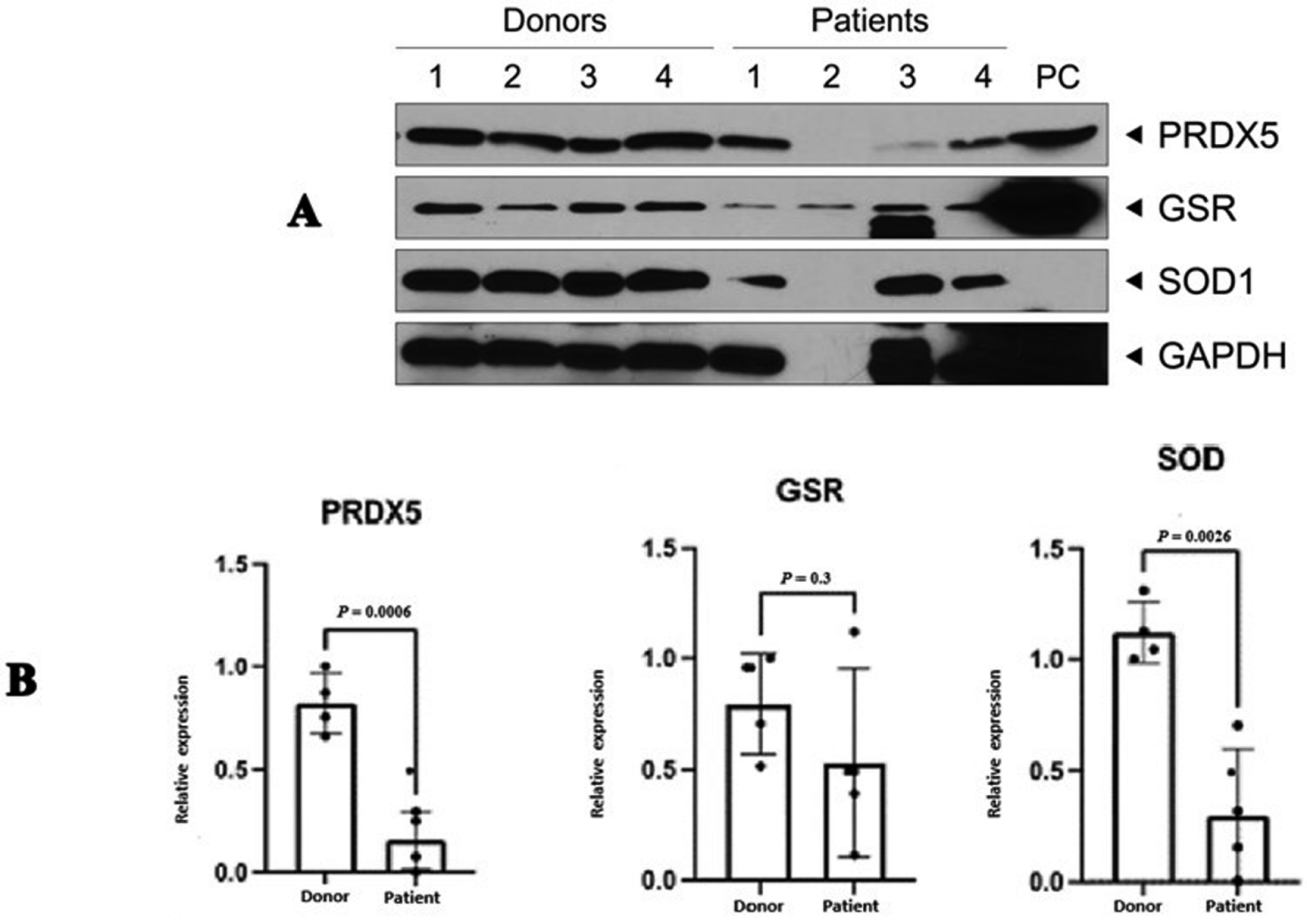
Western blot validation of three differentially expressed proteins in patients with idiopathic primary infertility (*n* = 4) and in healthy fertile donors (*n* = 4). **(A)** Representative image of WB, **(B)** Quantification of the WB results. Results are expressed as the mean fold change ± standard error of the mean and normalised to that of the control protein. PRDX5 and SOD were significantly decreased in patient sperm samples when compared with that of healthy donors. GAPDH. GSR, glutathione disulphide reductase; SOD, superoxide dismutase; PRDX5, peroxiredoxin-5; GAPDH, glyceraldehyde 3-phosphate dehydrogenase.

Analysis of the western blot results (Figure 3) revealed that two proteins, SOD2 (3.8 fold; *P* = 0.0026), and PRDX5 (5.3 fold; *P* = 0.0006), were expressed in accordance with that observed in the proteomic analysis (Table 5). No significant difference was observed in the expression of GSR (*P* = 0.3); however, this result is inconclusive considering the low concentration observed for the normalizing protein GAPDH as well as the increased expression of this protein in one of the four patient samples (Patient 2).

## Discussion

Idiopathic male infertility is diagnosed when there is an abnormality in the semen analysis with no obvious etiology. In this study, we identified mitochondrial dysfunction as a key factor linked to idiopathic primary male infertility. Using global proteomics analysis, we found a variety of DEPs between healthy donors and infertile patients, with a notable subset related to mitochondrial function. Specifically, three mitochondrial proteins (GSR, PRDX5, and SOD2) exhibited expression patterns that have not been previously documented in the context of male infertility. PRDX5 and SOD2 were significantly underexpressed, while GSR was overexpressed in the proteomics analysis, and bioinformatics indicated a general downregulation of mitochondrial activity in the infertile group. These results offer a potential basis for new biomarkers and therapeutic targets for diagnosing and managing idiopathic male infertility. The changes in expression of PRDX5 and SOD2, in particular, highlight the importance of oxidative stress regulation in this condition.

In sperm cells, these proteins work together to control oxidative stress. SOD2 initially neutralizes superoxide radicals, resulting in the formation of hydrogen peroxide (38). PRDX5 subsequently converts this hydrogen peroxide into water, which helps prevent the buildup of ROS (39). At the same time, GSR regenerates glutathione, maintaining the cell's antioxidant capacity (40). Any disruptions in the expression or function of these enzymes can lead to an imbalance in oxidative stress, raising ROS levels and damaging sperm DNA, lipids, and proteins, all of which can negatively impact sperm quality and fertility (41).

PRDX5 is a mitochondrial protein encoded by *PRDX5* in humans; it belongs to the six-member peroxiredoxin family of antioxidant enzymes. PRDX5 functions as a critical cytoprotective antioxidant enzyme that effectively mitigates the build-up of both endogenous and exogenous peroxides, including H_2_O_2_ (42). PRDX5 plays a crucial role in antioxidative and cytoprotective functions during oxidative stress. Reduced PRDX5 expression has been linked to an increased susceptibility to oxidative damage and peroxide-induced apoptosis (43) Previous proteomic studies have revealed decreased levels of PRDX5 in the spermatozoa of male patients with infertility (44). Abnormal PRDX5 activity affects various sperm-related factors, including motility, capacitation, mitochondrial membrane potential, and DNA integrity (45). These findings highlight the intricate roles of PRDX5 and its fellow peroxiredoxins in sperm function and overall male reproductive health. Our findings demonstrate for the first time, an association between PRDX5 expression and idiopathic primary male infertility.

SOD2 is encoded by *SOD2* in humans; it belongs to the superoxide dismutase iron/manganese family. SOD2 catalyzes the conversion of toxic superoxide, a by-product of the mitochondrial electron transport chain, into H_2_O_2_ and O_2_ (46). As a result, SOD2 effectively removes mitochondrial ROS, thereby protecting mitochondria and cells against oxidative stress. A clear connection exists between SOD gene polymorphisms and male infertility, with impacts on *in vitro* fertilization (IVF) outcomes (47). Our study further underscores the significance of SOD in the context of idiopathic primary male infertility using proteomic analysis. Similar to our findings regarding PRDX5, this finding emphasizes the potential role of oxidative stress as a causative factor in idiopathic primary male infertility owing to low protein levels of PRDX5 and SOD2.

GSR is encoded by *GSR* in humans; it catalyzes the reduction of glutathione disulphide to glutathione, a crucial molecule for combating oxidative stress and maintaining a cellular reducing environment (48). GSR has previously been reported as a biomarker of male infertility, particularly in cases associated with oxidative stress and varicoceles (49). According to our proteomics analysis, GSR was overexpressed (NSAF ratio = 4.43, *P* = 0.00070). However, IPA revealed a negative change in GSR expression, and the WB analysis revealed no significant difference in the total GSR expression, although increased GSR expression was detected in one of the four patient samples. Multiple factors could explain these differences, such as data normalization, data processing errors, control group-related differences, statistical analysis methods, and biological variability (46, 50, 51). Our data check did not reveal any technical or analytical errors; thus, we assume that the aforementioned differences in GSR expression are due to biological variability. This assumption needs to be validated using larger sample sizes for both the proteomics study and the western blot-based validation.

To the best of our knowledge, this study represents the first report on an association between PRDX5, and SOD2 expression and idiopathic primary male infertility. This indicates that the mitochondria in sperm of patients with idiopathic primary male infertility likely exhibit oxidative stress owing to relatively low antioxidant enzyme activity expanding our understanding of this condition beyond sperm parameters such as concentration, motility, and morphology. This association of idiopathic male infertility and mitochondrial oxidative stress was also indicated through the study by Agarwal et al. (52) when the researchers evaluated the sperm proteome of idiopathic infertile male before and after antioxidant supplementation, revealing post-treatment activation of transcriptional factors associated with antioxidant defence system and free radical scavenging.

The major limitations of this pilot study include its small sample size used for protein validation through western blot. We were able to include only 4 samples in each group and it turns out to be insufficient to validate the proteomics and bioinformatics analysis. Furthermore, although damage to sperm resulting in infertility, including DNA damage, caused by mitochondrial stress remains a key hypothesis, the mechanistic basis for these changes remains unknown, especially because we did not exclude patients with high levels of oxidative stress or high DNA fragmentation. These findings need to be re-evaluated in patients with primary infertility with and without high oxidative stress or high DNA fragmentation. Future studies should evaluate the response of the mtDNA repair mechanisms, as it could offer deeper insights into the underlying pathology of sperm damage. This could include measuring key enzymes involved in mtDNA repair, such as those in the base excision repair pathway, and assessing their correlation with mitochondrial protein dysfunction.

Whether these findings offer additional diagnostic insights beyond DNA damage alone remains to be tested, but they do potentially support the rapid assessment of key protein content using ELISA rather than complex DNA fragmentation testing. This study offers mitochondrial pathology as the underlying etiology of idiopathic male infertility and suggests an association with the downregulated expression of mitochondrial PRDX5 and SOD2. These findings encourage further mechanistic research and development of improved diagnostic techniques based on mitochondrial proteins for identifying mitochondrial pathology causing male infertility.

## Data Availability

The original contributions presented in the study are included in the article/Supplementary Material, further inquiries can be directed to the corresponding author.
