# Supplementary material for: Mitochondrial dysfunction signatures in idiopathic primary male infertility: a validated proteomics-based diagnostic approach
Source: Front Reprod Health. 2024 Dec 12;6:1479568. doi: 10.3389/frph.2024.1479568 (PMC11669654; doi:10.3389/frph.2024.1479568)
Supplement: Supplementary file 1 [file Table1.docx]

**Appendix. Supplementary Material A**

**Protein extraction and identification, proteomics analysis, LC-MS analysis, database searching, criteria for protein identification, and quantitative proteomics**

**Semen processing and protein extraction**

To ensure preservation of the semen samples, a gradual freezing protocol was implemented, utilizing TEST-yolk buffer (Irvine Scientific, Santa Ana, CA, USA). Following this, the samples were collected and stored at −80 °C for analysis.

For proteomics analysis, the samples were thawed at 37 °C for 20 min and then centrifuged at 4000 *g* for 10 min to separate the spermatozoa. The sperm cells were then subjected to a triple wash with phosphate-buffered saline (Irvine Scientific) and centrifuged again at 4000 *g* for 10 min at 4 °C. To initiate cell lysis, the sperm pellet was treated with Radio-immunoprecipitation assay (RIPA) buffer (Sigma-Aldrich, St. Louis, MO, USA), supplemented with Protease Inhibitor Cocktail, complete™ ULTRA Tablets, EDTA-free (Roche, Mannheim, Germany), at a ratio of 100 µl RIPA per million sperm. The mixture was incubated overnight at 4 °C. Following this, samples underwent centrifugation at 10,000 *g* for 30 min at 4 °C, and the resulting supernatant was transferred to a new centrifuge tube. Protein quantification was conducted using a Pierce BCA Protein Assay Kit (Thermo Fisher Scientific, Waltham, MA, USA) following the manufacturer's guidelines.

**Top of Form**

**Protein identification and global proteomics analysis**

Global proteomic analysis was conducted in triplicate for both patient and control groups and quantified using the label-free spectral counting method. Each sample (15 μg protein) underwent SDS-PAGE using a 12.5% Tris–HCl 1D gel. Electrophoresis was carried out at a constant 150 V for 35 min until the standard proteins reached one-third of the total length of the gel in preparation for subsequent GelC experiments. After electrophoresis, the gel was fixed in a 50% ethanol/10% acetic acid solution for 30 min, thoroughly washed with water, and stained with Coomassie blue.

For protein digestion, the entire gel lane was divided into six smaller gel pieces, which were washed and dehydrated in acetonitrile. The proteins in the gel fractions were reduced with dithiothreitol, alkylated with iodoacetamide, and then digested in-gel by adding 5 μL of 10 ng/μL trypsin in 50 mM ammonium bicarbonate. The fractions were left to incubate overnight at room temperature of 20-25°C to ensure complete digestion and then extracted from the gel using two aliquots (30 μL each) of 50% acetonitrile with 5% formic acid. Triplicate runs of gels using samples from fertile control and idiopathic infertility groups were performed to assess technical reproducibility of the assay.

Top of Form

Bottom of Form**Top of FormLiquid chromatography-mass spectrometry analysis (LC-MS)**

The protein extracts from the SDS-PAGE gel slides were combined and concentrated to a final volume of less than 10 μL using a Speedvac, and then resuspended in 1% acetic acid to achieve a final volume of approximately 30 μL for LC-MS analysis. The LC-MS system was a Finnigan LTQ-Orbitrap Elite hybrid mass spectrometer, Burladingen, Germany. We employed was a Dionex 15 cm × 75 μm internal diameter Acclaim Pepmap C18, 2 μm, 100 Å reversed-phase capillary chromatography HPLC column. Five μL volumes of the extract were injected and the peptides eluted from the column by an acetonitrile/0.1% formic acid gradient at a flow rate of 0.25μL/min were introduced into the source of the mass spectrometer on-line. The micro electrospray ion source was operated at 2.0 kV. Digestion was analysed using the data-dependent multitask capability of the instrument, acquiring full-scan mass spectra to determine peptide molecular weights, and tandem mass spectra (MS/MS) to elucidate the amino acid sequence in consecutive instrument scans.Top of Form

**Database searching**

Tandem mass spectra were extracted using Proteome Discoverer version 1.4.1.288 without charge state deconvolution or de-isotoping. Subsequently, all MS/MS samples were analysed using three search engines: Mascot (version 2.3.02, Matrix Science, London, UK), Sequest (version 1.4.0.288, Thermo Fisher Scientific), and X! Tandem [GPM, thegpm.org; version CYCLONE (2010.12.01.1)]. A Mascot, Sequest, and X! A tandem search was configured to search the human reference database, comprising 33,292 entries, while assuming trypsin digestion. These searches were conducted with a fragment ion mass tolerance of 1.0 Da and a parent ion tolerance of 10 parts per million. Fixed modifications of carbamidomethyl for cysteine and variable modifications for methionine oxidation were specified in the analytical settings.

**Top of Form**

**Criteria for protein identification**

To validate the identification of peptides and proteins based on the MS/MS data, we employed Scaffold (version 4.0.6.1, Proteome Software Inc., Portland, OR, USA). Peptide identifications were considered reliable if they achieved a probability greater than 95.0%, as determined by the Peptide Prophet algorithm (Eng et al., 1994), with the application of scaffold delta-mass correction. For protein identification, stringent criteria were applied, necessitating a probability exceeding 99.0% to maintain a false detection rate of less than 1.0% and a minimum of two identified peptides per protein. Protein probabilities were determined using a protein probe algorithm(Serang and Noble, 2012). When proteins contained similar peptides and could not be distinguished by MS/MS analysis alone, they were grouped together following the principles of parsimony. To enhance annotation, proteins were associated with Gene Ontology terms obtained from the National Centre for Biotechnology Information and downloaded on October 21, 2013.

**Top of FormTop of FormQuantitative proteomics**

For quantitative proteomic analysis, protein quantities were assessed by comparing the number of spectral counts (SpCs) used to identify each protein. SpCs represent the total number of mass spectral-matching peptides for a specific protein within a complex mixture. Before conducting relative protein quantification, we used the normalized spectral abundance factor (NSAF) approach(Zhang et al., 2010)to normalize the spectral counts. This method considers variations between replicate sample analyses and acknowledges that longer proteins often yield better peptide identification than do shorter proteins(Zybailov et al., 2005).

Differentially expressed proteins (DEPs) were identified using distinct criteria, including significance tests and fold-change thresholds guided by the average SpC of each protein from multiple runs. The accurate quantification and detection of genuine biological changes depend on the absolute SpC of a protein.

Recognizing that errors in proteomic analysis are more prevalent in proteins with lower abundance, we tailored our DEP filters based on the overall protein abundance. We applied varying constraints on the SpC levels to mitigate bias and maintain a consistent false-positive rate for all proteins.

Proteins were categorized as high medium ,low or very low in abundance based on their average spectral counts across three replicate runs. Distinct constraints for significance tests (*P*-value) and fold-change cutoffs (or NSAF ratio) were applied to these four abundance categories as outlined below.

1. Very low abundance: spectral count range 1.7–7; *P* ≤ 0.001 and NSAF ratio ≥2.5 for overexpressed and ≤0.4 for underexpressed proteins
2. Low abundance: spectral count range 8–19; *P* ≤ 0.01 and NSAF ratio ≥2.5 for overexpressed and ≤0.4 for underexpressed proteins
3. Medium abundance: spectral count range 20–79; *P* ≤ 0.05 and NSAF ratio ≥2.0 for overexpressed and ≤0.5 for underexpressed proteins
4. High abundance: spectral counts >80; *P* ≤ 0.05 and NSAF ratio ≥1.5 for overexpressed and ≤0.67 for underexpressed proteins

Applying varying statistical (i.e., *P*-value) and biological (i.e., fold change) constraints to the four defined categories (high, medium, low, and very low) based on spectral count levels ensured accurate quantification and detection of real biological changes. These criteria were established through control experiments that utilized the NSAF spectral counting method and analysis of two identical samples.**Bottom of Form**

**References**

Eng, J.K., McCormack, A.L., Yates, J.R., 1994. An approach to correlate tandem mass spectral data of peptides with amino acid sequences in a protein database. J Am Soc Mass Spectrom (5), 976–989. doi:10.1016/1044-0305(94)80016-2

Serang, O., Noble, W., 2012. A review of statistical methods for protein identification using tandem mass spectrometry. Stat Interface (5), 3–20. doi:10.4310/sii.2012.v5.n1.a2

Zhang, Y., Wen, Z., Washburn, M.P., Florens, L., 2010. Refinements to label free proteome quantitation: how to deal with peptides shared by multiple proteins. Anal Chem (82), 2272–2281. doi:10.1021/AC9023999

Zybailov, B., Coleman, M.K., Florens, L., Washburn, M.P., 2005. Correlation of relative abundance ratios derived from peptide ion chromatograms and spectrum counting for quantitative proteomic analysis using stable isotope labeling. Anal Chem (77), 6218–6224. doi:10.1021/AC050846R
