# Supplementary material for: Mitochondrial dysfunction signatures in idiopathic primary male infertility: a validated proteomics-based diagnostic approach
Source: Front Reprod Health. 2024 Dec 12;6:1479568. doi: 10.3389/frph.2024.1479568 (PMC11669654; doi:10.3389/frph.2024.1479568)
Supplement: Supplementary file 2 [file Table2.docx]

**Supplementary Material B .** Differentially expressed proteins (DEPs) with the normalized spectral abundance factor (NSAF) ratio.

Group1: Healthy fertile Donors

Group2: Patients with idiopathic infertility

| Protein | accession | Average SC | | Abundance | | NSAF ratio | T-Test | Expression |
| --- | --- | --- | --- | --- | --- | --- | --- | --- |
|  |  | Group 2 | Group 1 | Group 2 | Group 1 | Group2/Group1 | P-value |  |
| retinoid-inducible serine carboxypeptidase precursor | 11055992 | 0.0 | 7.7 | ni^*^ | VL | 0.00 | 2.848E-07 | Unique to Group 1 |
| EF-hand calcium-binding domain-containing protein 1 isoform a | 13375787 | 0.0 | 7.0 | ni | VL | 0.00 | 4.267E-07 | Unique to Group 1 |
| NADH dehydrogenase [ubiquinone] 1 beta subcomplex subunit 7 | 10764847 | 0.0 | 5.7 | ni | VL | 0.00 | 1.269E-06 | Unique to Group 1 |
| eukaryotic translation elongation factor 1 epsilon-1 isoform 1 | 4758862 | 0.0 | 6.3 | ni | VL | 0.00 | 4.769E-06 | Unique to Group 1 |
| acyl-coenzyme A thioesterase 13 isoform 2 | 231567183 | 0.0 | 5.3 | ni | VL | 0.00 | 2.140E-05 | Unique to Group 1 |
| ran-specific GTPase-activating protein | 4506407 | 0.0 | 5.3 | ni | VL | 0.00 | 2.603E-05 | Unique to Group 1 |
| A disintegrin and metalloproteinase with thrombospondin motifs 1 preproprotein | 50845384 | 0.0 | 6.7 | ni | VL | 0.00 | 0.00010 | Unique to Group 1 |
| actin-like protein 9 | 194097462 | 0.0 | 3.7 | ni | VL | 0.00 | 0.00011 | Unique to Group 1 |
| 3-mercaptopyruvate sulfurtransferase isoform 2 | 61835204 | 0.0 | 7.7 | ni | VL | 0.00 | 0.00012 | Unique to Group 1 |
| 60S ribosomal protein L12 | 4506597 | 0.0 | 6.3 | ni | VL | 0.00 | 0.00014 | Unique to Group 1 |
| uncharacterized protein C22orf43 | 56118955 | 0.0 | 5.3 | ni | VL | 0.00 | 0.00019 | Unique to Group 1 |
| myotrophin | 21956645 | 0.0 | 7.0 | ni | VL | 0.00 | 0.00020 | Unique to Group 1 |
| atlastin-3 | 45827806 | 0.0 | 5.7 | ni | VL | 0.00 | 0.00025 | Unique to Group 1 |
| nucleoporin p58/p45 isoform a | 30102928 | 0.0 | 5.7 | ni | VL | 0.00 | 0.00026 | Unique to Group 1 |
| NADH dehydrogenase [ubiquinone] iron-sulfur protein 5 | 4758790 | 0.0 | 3.3 | ni | VL | 0.00 | 0.00026 | Unique to Group 1 |
| nucleoporin NUP53 | 31982904 | 0.0 | 7.0 | ni | VL | 0.00 | 0.00027 | Unique to Group 1 |
| stress-induced-phosphoprotein 1 | 5803181 | 0.0 | 4.7 | ni | VL | 0.00 | 0.00029 | Unique to Group 1 |
| thioredoxin-dependent peroxide reductase, mitochondrial isoform b | 32483377 | 0.0 | 4.3 | ni | VL | 0.00 | 0.00029 | Unique to Group 1 |
| low molecular weight phosphotyrosine protein phosphatase isoform c | 4757714 | 0.0 | 4.0 | ni | VL | 0.00 | 0.00030 | Unique to Group 1 |
| ubiquitin thioesterase OTUB2 | 12962939 | 0.0 | 4.3 | ni | VL | 0.00 | 0.00036 | Unique to Group 1 |
| transmembrane emp24 domain-containing protein 4 precursor | 33457308 | 0.0 | 4.3 | ni | VL | 0.00 | 0.00037 | Unique to Group 1 |
| protein FAM162A | 49355721 | 0.0 | 4.7 | ni | VL | 0.00 | 0.00041 | Unique to Group 1 |
| nucleosome assembly protein 1-like 4 | 5174613 | 0.0 | 6.7 | ni | VL | 0.00 | 0.00046 | Unique to Group 1 |
| uncharacterized protein C9orf135 | 58219541 | 0.0 | 6.0 | ni | VL | 0.00 | 0.00063 | Unique to Group 1 |
| SPRY domain-containing protein 7 isoform 1 | 20531765 | 0.0 | 3.7 | ni | VL | 0.00 | 0.00065 | Unique to Group 1 |
| sulfatase-modifying factor 2 isoform e precursor | 194248090 | 0.0 | 6.0 | ni | VL | 0.00 | 0.00072 | Unique to Group 1 |
| thiosulfate sulfurtransferase/rhodanese-like domain-containing protein 1 isoform 2 | 163965375 | 0.0 | 4.3 | ni | VL | 0.00 | 0.00075 | Unique to Group 1 |
| aspartyl-tRNA synthetase, cytoplasmic | 45439306 | 0.0 | 4.3 | ni | VL | 0.00 | 0.00075 | Unique to Group 1 |
| V-type proton ATPase subunit E 1 isoform a | 4502317 | 0.0 | 6.0 | ni | VL | 0.00 | 0.00075 | Unique to Group 1 |
| Sjoegren syndrome nuclear autoantigen 1 | 189571687 | 0.0 | 6.3 | ni | VL | 0.00 | 0.00094 | Unique to Group 1 |
| 26S protease regulatory subunit 7 isoform 1 | 4506209 | 0.0 | 13.3 | ni | L | 0.00 | 0.00000 | Unique to Group 1 |
| 26S protease regulatory subunit 4 | 24430151 | 0.0 | 18.3 | ni | L | 0.00 | 0.00000 | Unique to Group 1 |
| heterogeneous nuclear ribonucleoprotein M isoform a | 14141152 | 0.0 | 10.3 | ni | L | 0.00 | 0.00000 | Unique to Group 1 |
| calpain small subunit 1 | 51599151 | 0.0 | 16.7 | ni | L | 0.00 | 0.00000 | Unique to Group 1 |
| transmembrane and coiled-coil domain-containing protein 2 | 56847610 | 0.0 | 12.3 | ni | L | 0.00 | 0.00000 | Unique to Group 1 |
| enkurin | 21450721 | 0.0 | 13.0 | ni | L | 0.00 | 0.00001 | Unique to Group 1 |
| coiled-coil-helix-coiled-coil-helix domain-containing protein 3, mitochondrial precursor | 8923390 | 0.0 | 18.3 | ni | L | 0.00 | 0.00002 | Unique to Group 1 |
| 26S protease regulatory subunit 6B isoform 1 | 5729991 | 0.0 | 18.0 | ni | L | 0.00 | 0.00002 | Unique to Group 1 |
| kinectin isoform c | 118498368 | 0.0 | 8.3 | ni | L | 0.00 | 0.00002 | Unique to Group 1 |
| protein S100-A11 | 5032057 | 0.0 | 12.7 | ni | L | 0.00 | 0.00004 | Unique to Group 1 |
| outer dense fiber protein 3 | 19526475 | 0.0 | 15.3 | ni | L | 0.00 | 0.00004 | Unique to Group 1 |
| uncharacterized protein C9orf171 | 46409466 | 0.0 | 8.0 | ni | L | 0.00 | 0.00006 | Unique to Group 1 |
| dnaJ homolog subfamily B member 8 | 23503241 | 0.0 | 10.3 | ni | L | 0.00 | 0.00009 | Unique to Group 1 |
| endophilin-B1 isoform 2 | 331284170 | 0.0 | 18.0 | ni | L | 0.00 | 0.00009 | Unique to Group 1 |
| ATP synthase subunit gamma, mitochondrial isoform L (liver) precursor | 50345988 | 0.0 | 11.7 | ni | L | 0.00 | 0.00009 | Unique to Group 1 |
| rho GDP-dissociation inhibitor 1 isoform a | 4757768 | 0.0 | 16.0 | ni | L | 0.00 | 0.00009 | Unique to Group 1 |
| actin, alpha cardiac muscle 1 proprotein | 4885049 | 0.0 | 11.3 | ni | L | 0.00 | 0.00012 | Unique to Group 1 |
| eukaryotic translation initiation factor 3 subunit F | 4503519 | 0.0 | 13.0 | ni | L | 0.00 | 0.00013 | Unique to Group 1 |
| nuclear pore glycoprotein p62 | 24497605 | 0.0 | 13.7 | ni | L | 0.00 | 0.00016 | Unique to Group 1 |
| 60S ribosomal protein L7a | 4506661 | 0.0 | 8.3 | ni | L | 0.00 | 0.00016 | Unique to Group 1 |
| 2-oxoglutarate dehydrogenase, mitochondrial isoform 1 precursor | 51873036 | 0.0 | 18.3 | ni | L | 0.00 | 0.00018 | Unique to Group 1 |
| myosin regulatory light chain 12B | 15809016 | 0.0 | 13.0 | ni | L | 0.00 | 0.00020 | Unique to Group 1 |
| ubiquitin carboxyl-terminal hydrolase isozyme L3 | 5174741 | 0.0 | 19.7 | ni | L | 0.00 | 0.00020 | Unique to Group 1 |
| speriolin isoform 1 | 197276668 | 0.0 | 9.7 | ni | L | 0.00 | 0.00020 | Unique to Group 1 |
| adenylate kinase 8 | 22749187 | 0.0 | 16.0 | ni | L | 0.00 | 0.00023 | Unique to Group 1 |
| pro-cathepsin H preproprotein | 23110955 | 0.0 | 9.3 | ni | L | 0.00 | 0.00025 | Unique to Group 1 |
| nucleobindin-2 precursor | 4826870 | 0.0 | 18.7 | ni | L | 0.00 | 0.00026 | Unique to Group 1 |
| prenylated Rab acceptor protein 1 | 222144309 | 0.0 | 9.7 | ni | L | 0.00 | 0.00027 | Unique to Group 1 |
| annexin A11 | 22165431 | 0.0 | 10.0 | ni | L | 0.00 | 0.00034 | Unique to Group 1 |
| apoptosis-inducing factor 1, mitochondrial isoform 2 precursor | 22202629 | 0.0 | 8.3 | ni | L | 0.00 | 0.00040 | Unique to Group 1 |
| 26S proteasome non-ATPase regulatory subunit 3 | 25777612 | 0.0 | 13.7 | ni | L | 0.00 | 0.00042 | Unique to Group 1 |
| eukaryotic translation initiation factor 3 subunit I | 4503513 | 0.0 | 9.7 | ni | L | 0.00 | 0.00050 | Unique to Group 1 |
| rab GDP dissociation inhibitor beta isoform 1 | 6598323 | 0.0 | 19.3 | ni | L | 0.00 | 0.00051 | Unique to Group 1 |
| eukaryotic initiation factor 4A-II | 83700235 | 0.0 | 10.7 | ni | L | 0.00 | 0.00059 | Unique to Group 1 |
| dynein heavy chain 8, axonemal | 332688227 | 0.0 | 12.0 | ni | L | 0.00 | 0.00062 | Unique to Group 1 |
| enoyl-CoA delta isomerase 1, mitochondrial isoform 1 precursor | 62530384 | 0.0 | 16.3 | ni | L | 0.00 | 0.00063 | Unique to Group 1 |
| acrosome formation-associated factor isoform 1 | 239582757 | 0.0 | 13.3 | ni | L | 0.00 | 0.00068 | Unique to Group 1 |
| UBX domain-containing protein 11 isoform 2 | 116734679 | 0.0 | 9.7 | ni | L | 0.00 | 0.00075 | Unique to Group 1 |
| adenylate kinase 7 | 148727333 | 0.0 | 14.3 | ni | L | 0.00 | 0.00084 | Unique to Group 1 |
| mitochondrial import receptor subunit TOM22 homolog | 9910382 | 0.0 | 13.3 | ni | L | 0.00 | 0.00087 | Unique to Group 1 |
| signal peptidase complex catalytic subunit SEC11A | 7657609 | 0.0 | 8.3 | ni | L | 0.00 | 0.00098 | Unique to Group 1 |
| 14-3-3 protein beta/alpha | 21328448 | 0.0 | 8.0 | ni | L | 0.00 | 0.00104 | Unique to Group 1 |
| NADH dehydrogenase [ubiquinone] flavoprotein 2, mitochondrial precursor | 222080062 | 0.0 | 14.7 | ni | L | 0.00 | 0.00115 | Unique to Group 1 |
| voltage-dependent anion-selective channel protein 1 | 4507879 | 0.0 | 8.7 | ni | L | 0.00 | 0.00120 | Unique to Group 1 |
| dynactin subunit 1 isoform 3 | 205277392 | 0.0 | 9.7 | ni | L | 0.00 | 0.00151 | Unique to Group 1 |
| signal peptidase complex subunit 3 | 11345462 | 0.0 | 10.7 | ni | L | 0.00 | 0.00152 | Unique to Group 1 |
| dnaJ homolog subfamily B member 11 precursor | 7706495 | 0.0 | 8.7 | ni | L | 0.00 | 0.00208 | Unique to Group 1 |
| leucine zipper transcription factor-like protein 1 | 9966793 | 0.0 | 15.3 | ni | L | 0.00 | 0.00217 | Unique to Group 1 |
| isocitrate dehydrogenase [NAD] subunit beta, mitochondrial isoform a precursor | 28178821 | 0.0 | 14.7 | ni | L | 0.00 | 0.00231 | Unique to Group 1 |
| flavin reductase (NADPH) | 4502419 | 0.0 | 8.0 | ni | L | 0.00 | 0.00258 | Unique to Group 1 |
| transmembrane protein 190 precursor | 21040263 | 0.0 | 11.7 | ni | L | 0.00 | 0.00270 | Unique to Group 1 |
| ropporin-1-like protein | 17572807 | 0.0 | 15.7 | ni | L | 0.00 | 0.00275 | Unique to Group 1 |
| histone H2A-Bbd type 2/3 | 63029935 | 0.0 | 9.7 | ni | L | 0.00 | 0.00281 | Unique to Group 1 |
| dnaJ homolog subfamily B member 1 | 5453690 | 0.0 | 9.0 | ni | L | 0.00 | 0.00282 | Unique to Group 1 |
| melanoma inhibitory activity protein 3 precursor | 122891870 | 0.0 | 12.3 | ni | L | 0.00 | 0.00291 | Unique to Group 1 |
| endoplasmic reticulum resident protein 29 isoform 1 precursor | 5803013 | 0.0 | 18.7 | ni | L | 0.00 | 0.00292 | Unique to Group 1 |
| slit homolog 2 protein precursor | 4759146 | 0.0 | 9.7 | ni | L | 0.00 | 0.00301 | Unique to Group 1 |
| 14-3-3 protein sigma | 5454052 | 0.0 | 14.0 | ni | L | 0.00 | 0.00329 | Unique to Group 1 |
| cytochrome b-c1 complex subunit 7 isoform 1 | 5454152 | 0.0 | 14.0 | ni | L | 0.00 | 0.00353 | Unique to Group 1 |
| 26S proteasome non-ATPase regulatory subunit 14 | 5031981 | 0.0 | 15.7 | ni | L | 0.00 | 0.00395 | Unique to Group 1 |
| thioredoxin isoform 1 | 50592994 | 0.0 | 8.7 | ni | L | 0.00 | 0.00397 | Unique to Group 1 |
| GTP-binding nuclear protein Ran | 5453555 | 0.0 | 13.3 | ni | L | 0.00 | 0.00437 | Unique to Group 1 |
| T-complex protein 11 homolog isoform 1 | 148226214 | 0.0 | 9.3 | ni | L | 0.00 | 0.00458 | Unique to Group 1 |
| dynactin subunit 2 | 5453629 | 0.0 | 13.3 | ni | L | 0.00 | 0.00497 | Unique to Group 1 |
| tetratricopeptide repeat protein 25 | 13899233 | 0.0 | 10.3 | ni | L | 0.00 | 0.00518 | Unique to Group 1 |
| programmed cell death protein 6 | 7019485 | 0.0 | 8.3 | ni | L | 0.00 | 0.00522 | Unique to Group 1 |
| dnaJ homolog subfamily A member 2 | 5031741 | 0.0 | 10.7 | ni | L | 0.00 | 0.00571 | Unique to Group 1 |
| transcription factor A, mitochondrial precursor | 4507401 | 0.0 | 8.3 | ni | L | 0.00 | 0.00596 | Unique to Group 1 |
| 26S proteasome non-ATPase regulatory subunit 2 | 25777602 | 0.0 | 16.3 | ni | L | 0.00 | 0.00618 | Unique to Group 1 |
| pyruvate dehydrogenase protein X component, mitochondrial isoform 2 | 203098816 | 0.0 | 9.3 | ni | L | 0.00 | 0.00644 | Unique to Group 1 |
| cytochrome c | 11128019 | 0.0 | 11.0 | ni | L | 0.00 | 0.00652 | Unique to Group 1 |
| protein FAM154A | 301129242 | 0.0 | 9.7 | ni | L | 0.00 | 0.00652 | Unique to Group 1 |
| barrier-to-autointegration factor | 4502389 | 0.0 | 19.0 | ni | L | 0.00 | 0.00660 | Unique to Group 1 |
| cAMP-dependent protein kinase catalytic subunit alpha isoform 2 | 46909584 | 0.0 | 12.7 | ni | L | 0.00 | 0.00783 | Unique to Group 1 |
| diablo homolog, mitochondrial isoform 1 precursor | 9845297 | 0.0 | 9.0 | ni | L | 0.00 | 0.00798 | Unique to Group 1 |
| adenylate kinase 2, mitochondrial isoform a | 4502013 | 0.0 | 8.7 | ni | L | 0.00 | 0.00803 | Unique to Group 1 |
| bifunctional aminoacyl-tRNA synthetase | 62241042 | 0.0 | 10.3 | ni | L | 0.00 | 0.00816 | Unique to Group 1 |
| thioredoxin-related transmembrane protein 4 precursor | 0 | 0.0 | 19.7 | ni | L | 0.00 | 0.00860 | Unique to Group 1 |
| cytochrome b-c1 complex subunit Rieske, mitochondrial | 163644321 | 0.0 | 14.3 | ni | L | 0.00 | 0.00931 | Unique to Group 1 |
| izumo sperm-egg fusion protein 2 precursor | 63999117 | 0.0 | 9.7 | ni | L | 0.00 | 0.00999 | Unique to Group 1 |
| prohibitin | 4505773 | 0.0 | 22.3 | ni | M | 0.00 | 0.00000 | Unique to Group 1 |
| reticulocalbin-2 precursor | 4506457 | 0.0 | 23.7 | ni | M | 0.00 | 0.00000 | Unique to Group 1 |
| 26S protease regulatory subunit 6A | 21361144 | 0.0 | 31.0 | ni | M | 0.00 | 0.00000 | Unique to Group 1 |
| abhydrolase domain-containing protein 10, mitochondrial precursor | 8923001 | 0.0 | 24.3 | ni | M | 0.00 | 0.00001 | Unique to Group 1 |
| pyruvate dehydrogenase E1 component subunit alpha, testis-specific form, mitochondrial precursor | 4885543 | 0.0 | 20.3 | ni | M | 0.00 | 0.00001 | Unique to Group 1 |
| trifunctional enzyme subunit beta, mitochondrial precursor | 4504327 | 0.0 | 74.0 | ni | M | 0.00 | 0.00001 | Unique to Group 1 |
| annexin A3 | 4826643 | 0.0 | 37.7 | ni | M | 0.00 | 0.00002 | Unique to Group 1 |
| lysosomal alpha-glucosidase preproprotein | 119393891 | 0.0 | 25.0 | ni | M | 0.00 | 0.00009 | Unique to Group 1 |
| protein disulfide-isomerase A4 precursor | 4758304 | 0.0 | 50.0 | ni | M | 0.00 | 0.00010 | Unique to Group 1 |
| dolichyl-diphosphooligosaccharide--protein glycosyltransferase 48 subunit precursor | 20070197 | 0.0 | 30.7 | ni | M | 0.00 | 0.00015 | Unique to Group 1 |
| nucleoporin p54 | 26051237 | 0.0 | 30.0 | ni | M | 0.00 | 0.00017 | Unique to Group 1 |
| protein FAM166A | 48717426 | 0.0 | 37.7 | ni | M | 0.00 | 0.00029 | Unique to Group 1 |
| radial spoke head protein 9 homolog isoform 1 | 32964825 | 0.0 | 29.7 | ni | M | 0.00 | 0.00036 | Unique to Group 1 |
| radial spoke head protein 3 homolog | 31543559 | 0.0 | 24.3 | ni | M | 0.00 | 0.00049 | Unique to Group 1 |
| NADH dehydrogenase [ubiquinone] iron-sulfur protein 3, mitochondrial precursor | 4758788 | 0.0 | 31.0 | ni | M | 0.00 | 0.00077 | Unique to Group 1 |
| radial spoke head protein 6 homolog A | 13540559 | 0.0 | 65.3 | ni | M | 0.00 | 0.00112 | Unique to Group 1 |
| mitochondrial inner membrane protein isoform 1 | 154354964 | 0.0 | 53.0 | ni | M | 0.00 | 0.00116 | Unique to Group 1 |
| myeloid leukemia factor 1 isoform 1 | 11967975 | 0.0 | 30.7 | ni | M | 0.00 | 0.00120 | Unique to Group 1 |
| isocitrate dehydrogenase [NAD] subunit alpha, mitochondrial precursor | 5031777 | 0.0 | 29.3 | ni | M | 0.00 | 0.00135 | Unique to Group 1 |
| alpha-centractin | 5031569 | 0.0 | 41.3 | ni | M | 0.00 | 0.00144 | Unique to Group 1 |
| 26S proteasome non-ATPase regulatory subunit 7 | 25777615 | 0.0 | 20.7 | ni | M | 0.00 | 0.00202 | Unique to Group 1 |
| sperm-associated antigen 6 isoform 1 | 6912678 | 0.0 | 23.3 | ni | M | 0.00 | 0.00215 | Unique to Group 1 |
| enoyl-CoA hydratase, mitochondrial | 194097323 | 0.0 | 26.7 | ni | M | 0.00 | 0.00222 | Unique to Group 1 |
| ATP synthase subunit O, mitochondrial precursor | 4502303 | 0.0 | 30.7 | ni | M | 0.00 | 0.00279 | Unique to Group 1 |
| stomatin-like protein 2 | 7305503 | 0.0 | 25.0 | ni | M | 0.00 | 0.00287 | Unique to Group 1 |
| heme oxygenase 2 | 8051608 | 0.0 | 21.3 | ni | M | 0.00 | 0.00310 | Unique to Group 1 |
| uncharacterized protein C9orf9 | 33285006 | 0.0 | 23.0 | ni | M | 0.00 | 0.00431 | Unique to Group 1 |
| nucleoside diphosphate kinase homolog 5 | 4505413 | 0.0 | 28.3 | ni | M | 0.00 | 0.00434 | Unique to Group 1 |
| isochorismatase domain-containing protein 2, mitochondrial isoform 2 | 13376007 | 0.0 | 20.7 | ni | M | 0.00 | 0.00865 | Unique to Group 1 |
| ATP synthase subunit b, mitochondrial precursor | 21361565 | 0.0 | 21.7 | ni | M | 0.00 | 0.01209 | Unique to Group 1 |
| coiled-coil domain-containing protein 147 | 56961680 | 0.0 | 20.7 | ni | M | 0.00 | 0.02850 | Unique to Group 1 |
| calcium-binding tyrosine phosphorylation-regulated protein isoform c | 24797112 | 1.7 | 146.3 | VL | H | 0.01 | 0.00030 | UE |
| acetyl-CoA acetyltransferase, mitochondrial precursor | 4557237 | 1.7 | 141.7 | VL | H | 0.02 | 0.00006 | UE |
| neutral alpha-glucosidase AB isoform 2 precursor | 38202257 | 1.0 | 50.3 | VL | M | 0.02 | 0.00238 | UE |
| NADH-ubiquinone oxidoreductase 75 subunit, mitochondrial isoform 5 | 316983160 | 1.7 | 49.7 | VL | M | 0.03 | 0.00074 | UE |
| hypoxia up-regulated protein 1 precursor | 5453832 | 5.7 | 152.0 | VL | H | 0.05 | 0.00194 | UE |
| hydroxyacyl-coenzyme A dehydrogenase, mitochondrial isoform 1 precursor | 296179427 | 0.7 | 26.3 | VL | M | 0.05 | 0.00654 | UE |
| elongation factor 1-delta isoform 1 | 304555581 | 1.3 | 37.7 | VL | M | 0.05 | 0.00226 | UE |
| uncharacterized protein C1orf56 precursor | 20149646 | 4.0 | 142.3 | VL | H | 0.05 | 0.00008 | UE |
| sperm surface protein Sp17 | 8394343 | 1.7 | 47.0 | VL | M | 0.05 | 0.00058 | UE |
| pyruvate dehydrogenase E1 component subunit beta, mitochondrial isoform 1 precursor | 156564403 | 2.3 | 78.7 | VL | M | 0.05 | 0.00010 | UE |
| 26S proteasome non-ATPase regulatory subunit 13 isoform 1 | 157502193 | 0.7 | 24.3 | VL | M | 0.05 | 0.00122 | UE |
| importin subunit alpha-2 | 4504897 | 0.7 | 25.0 | VL | M | 0.05 | 0.00008 | UE |
| LETM1 and EF-hand domain-containing protein 1, mitochondrial precursor | 6912482 | 1.0 | 11.0 | VL | L | 0.06 | 0.00111 | UE |
| lysyl-tRNA synthetase isoform 1 | 194272210 | 0.7 | 9.7 | VL | L | 0.06 | 0.00078 | UE |
| exportin-7 | 154448892 | 1.0 | 11.3 | VL | L | 0.06 | 0.00067 | UE |
| myosin light polypeptide 6 isoform 2 | 88999583 | 2.3 | 41.0 | VL | M | 0.06 | 0.00006 | UE |
| dolichyl-diphosphooligosaccharide--protein glycosyltransferase subunit 2 isoform 2 precursor | 209413738 | 1.3 | 30.7 | VL | M | 0.07 | 0.02049 | UE |
| nuclear pore complex protein Nup93 isoform 1 | 208609990 | 1.0 | 22.3 | VL | M | 0.07 | 0.00200 | UE |
| protein phosphatase 1 regulatory subunit 7 | 4506013 | 1.7 | 43.7 | VL | M | 0.07 | 0.00049 | UE |
| calmegin precursor | 4758004 | 1.3 | 11.7 | VL | L | 0.08 | 0.00079 | UE |
| cAMP-dependent protein kinase type I-alpha regulatory subunit | 4506063 | 1.3 | 39.7 | VL | M | 0.08 | 0.00654 | UE |
| extracellular matrix protein 1 isoform 3 precursor | 322302700 | 1.0 | 11.3 | VL | L | 0.08 | 0.00668 | UE |
| protein disulfide-isomerase A6 precursor | 5031973 | 1.7 | 48.3 | VL | M | 0.09 | 0.00216 | UE |
| valyl-tRNA synthetase | 5454158 | 6.3 | 56.3 | VL | M | 0.09 | 0.00282 | UE |
| beta-2-microglobulin precursor | 4757826 | 2.7 | 20.7 | VL | M | 0.09 | 0.00083 | UE |
| glypican-1 precursor | 167001141 | 1.7 | 13.0 | VL | L | 0.10 | 0.00108 | UE |
| acrosin-binding protein precursor | 17999524 | 8.3 | 98.0 | L | H | 0.11 | 0.00002 | UE |
| transmembrane emp24 domain-containing protein 9 precursor | 39725636 | 1.0 | 16.3 | VL | L | 0.12 | 0.00330 | UE |
| actin-related protein T2 | 29893808 | 0.7 | 24.7 | VL | M | 0.12 | 0.01584 | UE |
| mitochondria-eating protein | 21687119 | 1.7 | 23.0 | VL | M | 0.12 | 0.00326 | UE |
| alanyl-tRNA editing protein Aarsd1 isoform 1 | 217416402 | 1.0 | 15.3 | VL | L | 0.12 | 0.00264 | UE |
| annexin A5 | 4502107 | 6.7 | 102.3 | VL | H | 0.13 | 0.00007 | UE |
| synaptojanin-2-binding protein | 157388993 | 2.0 | 19.7 | VL | L | 0.13 | 0.00550 | UE |
| 3-hydroxyisobutyrate dehydrogenase, mitochondrial precursor | 23308751 | 4.0 | 34.3 | VL | M | 0.14 | 0.01785 | UE |
| stromal cell-derived factor 2-like protein 1 precursor | 56243533 | 0.7 | 8.3 | VL | L | 0.14 | 0.00461 | UE |
| transcription elongation factor B polypeptide 1 isoform a | 325652033 | 1.3 | 9.7 | VL | L | 0.16 | 0.00701 | UE |
| calcium-binding tyrosine phosphorylation-regulated protein isoform a | 24797108 | 7.0 | 54.3 | VL | M | 0.17 | 0.00248 | UE |
| annexin A2 isoform 2 | 209862831 | 6.7 | 121.7 | VL | H | 0.17 | 0.00018 | UE |
| alpha-actinin-4 | 12025678 | 8.0 | 40.3 | L | M | 0.17 | 0.00077 | UE |
| nuclear pore complex protein Nup155 isoform 1 | 24430149 | 6.0 | 31.7 | VL | M | 0.18 | 0.00119 | UE |
| leucine-rich repeat-containing protein 37B precursor | 53829385 | 17.3 | 141.3 | L | H | 0.18 | 0.00020 | UE |
| erlin-2 isoform 1 | 6005721 | 5.0 | 57.7 | VL | M | 0.18 | 0.00001 | UE |
| dynein heavy chain 17, axonemal | 256542310 | 3.0 | 12.0 | VL | L | 0.18 | 0.00155 | UE |
| protein disulfide-isomerase A3 precursor | 21361657 | 17.3 | 229.3 | L | H | 0.19 | 0.00021 | UE |
| ATP synthase subunit beta, mitochondrial precursor | 32189394 | 28.3 | 336.7 | M | H | 0.19 | 0.00004 | UE |
| dolichyl-diphosphooligosaccharide--protein glycosyltransferase subunit 1 precursor | 4506675 | 8.0 | 47.7 | L | M | 0.20 | 0.00474 | UE |
| calnexin precursor | 10716563 | 6.3 | 48.0 | VL | M | 0.20 | 0.00230 | UE |
| T-complex protein 1 subunit zeta-2 isoform 1 | 58331173 | 3.3 | 29.3 | VL | M | 0.22 | 0.02401 | UE |
| methionyl-tRNA synthetase, cytoplasmic | 14043022 | 2.7 | 8.7 | VL | L | 0.23 | 0.00572 | UE |
| peroxiredoxin-5, mitochondrial isoform a precursor | 6912238 | 6.3 | 48.3 | VL | M | 0.23 | 0.02573 | UE |
| cAMP-dependent protein kinase type II-alpha regulatory subunit | 4758958 | 6.3 | 60.7 | VL | M | 0.24 | 0.01518 | UE |
| delta(3,5)-Delta(2,4)-dienoyl-CoA isomerase, mitochondrial precursor | 70995211 | 3.0 | 26.0 | VL | M | 0.24 | 0.04520 | UE |
| transmembrane emp24 domain-containing protein 10 precursor | 98986464 | 2.7 | 20.3 | VL | M | 0.25 | 0.01029 | UE |
| importin subunit beta-1 | 19923142 | 8.7 | 35.3 | L | M | 0.26 | 0.03018 | UE |
| matrix-remodeling-associated protein 5 precursor | 139948432 | 7.3 | 26.7 | VL | M | 0.27 | 0.00639 | UE |
| voltage-dependent anion-selective channel protein 2 isoform 2 | 296317339 | 4.7 | 34.7 | VL | M | 0.27 | 0.02909 | UE |
| ATP synthase subunit d, mitochondrial isoform a | 5453559 | 4.3 | 30.3 | VL | M | 0.27 | 0.01907 | UE |
| axonemal dynein light intermediate polypeptide 1 | 37595560 | 4.0 | 27.7 | VL | M | 0.29 | 0.00043 | UE |
| adenylate kinase isoenzyme 1 | 4502011 | 4.0 | 42.0 | VL | M | 0.29 | 0.02797 | UE |
| heat shock cognate 71 protein isoform 1 | 5729877 | 21.3 | 93.3 | M | H | 0.30 | 0.00202 | UE |
| aconitate hydratase, mitochondrial precursor | 4501867 | 24.3 | 104.3 | M | H | 0.30 | 0.00137 | UE |
| ruvB-like 2 | 5730023 | 18.0 | 161.7 | L | H | 0.31 | 0.00024 | UE |
| heat shock protein HSP 90-alpha isoform 1 | 153792590 | 89.3 | 375.0 | H | H | 0.32 | 0.00002 | UE |
| heat shock protein HSP 90-beta | 20149594 | 23.3 | 76.7 | L | M | 0.33 | 0.01732 | UE |
| myosin-9 | 12667788 | 48.0 | 129.7 | L | H | 0.33 | 0.00673 | UE |
| fumarate hydratase, mitochondrial | 19743875 | 17.3 | 119.3 | L | H | 0.33 | 0.00024 | UE |
| 78 glucose-regulated protein precursor | 16507237 | 91.3 | 345.0 | H | H | 0.34 | 0.00086 | UE |
| cathelicidin antimicrobial peptide preproprotein | 348041314 | 15.7 | 81.7 | L | H | 0.34 | 0.01159 | UE |
| endoplasmin precursor | 4507677 | 73.3 | 268.7 | M | H | 0.35 | 0.00491 | UE |
| T-complex protein 1 subunit zeta isoform a | 4502643 | 5.7 | 30.7 | VL | M | 0.35 | 0.00645 | UE |
| sperm acrosome membrane-associated protein 1 precursor | 13569934 | 27.3 | 105.7 | M | H | 0.35 | 0.00007 | UE |
| leucine-rich repeat-containing protein 37A precursor | 289547512 | 18.3 | 82.0 | L | H | 0.38 | 0.01552 | UE |
| epididymal sperm-binding protein 1 precursor | 301601648 | 4.0 | 20.3 | VL | M | 0.40 | 0.03928 | UE |
| ADP-ribosylation factor 1 | 4502201 | 6.0 | 22.3 | VL | M | 0.40 | 0.01678 | UE |
| hexokinase-1 isoform HKI-ta/tb | 15991831 | 33.7 | 93.3 | M | H | 0.41 | 0.01448 | UE |
| heat shock 70 protein 1A/1B | 194248072 | 23.7 | 88.7 | M | H | 0.41 | 0.00150 | UE |
| heat shock-related 70 protein 2 | 13676857 | 139.0 | 507.7 | H | H | 0.43 | 0.00051 | UE |
| cytochrome c oxidase subunit 5B, mitochondrial precursor | 17017988 | 7.0 | 21.7 | VL | M | 0.43 | 0.00109 | UE |
| ATP synthase subunit alpha, mitochondrial precursor | 4757810 | 31.7 | 148.0 | M | H | 0.44 | 0.00791 | UE |
| heat shock 70 protein 4L | 31541941 | 14.7 | 39.7 | L | M | 0.45 | 0.00452 | UE |
| cytochrome c oxidase subunit 4 isoform 1, mitochondrial precursor | 4502981 | 15.0 | 45.0 | L | M | 0.47 | 0.02616 | UE |
| protein disulfide-isomerase precursor | 20070125 | 11.7 | 58.3 | L | M | 0.47 | 0.00063 | UE |
| 14-3-3 protein epsilon | 5803225 | 12.3 | 60.3 | L | M | 0.47 | 0.00640 | UE |
| T-complex protein 1 subunit beta isoform 1 | 5453603 | 23.0 | 126.0 | M | H | 0.48 | 0.00157 | UE |
| A-kinase anchor protein 3 | 21493041 | 113.7 | 342.7 | H | H | 0.48 | 0.00211 | UE |
| carboxypeptidase Z isoform 2 precursor | 62388875 | 8.0 | 21.3 | L | M | 0.49 | 0.01871 | UE |
| transitional endoplasmic reticulum ATPase | 6005942 | 50.3 | 102.3 | M | H | 0.54 | 0.04926 | UE |
| trifunctional enzyme subunit alpha, mitochondrial precursor | 20127408 | 34.7 | 84.7 | M | H | 0.55 | 0.01375 | UE |
| heat shock 70 protein 1-like | 124256496 | 42.7 | 122.7 | M | H | 0.55 | 0.00655 | UE |
| ruvB-like 1 | 4506753 | 28.7 | 107.7 | M | H | 0.56 | 0.01223 | UE |
| cytochrome b-c1 complex subunit 2, mitochondrial precursor | 50592988 | 18.0 | 90.0 | M | H | 0.56 | 0.02465 | UE |
| tubulin beta-4B chain | 5174735 | 195.7 | 675.7 | H | H | 0.57 | 0.00963 | UE |
| semenogelin-1 preproprotein | 4506883 | 395.7 | 902.3 | H | H | 0.64 | 0.01932 | UE |
| tubulin alpha-3C/D chain | 17921993 | 144.0 | 432.7 | H | H | 0.65 | 0.01942 | UE |
| triosephosphate isomerase isoform 2 | 226529917 | 150.3 | 159.3 | H | H | 1.69 | 0.00822 | OE |
| aminopeptidase N precursor | 157266300 | 127.7 | 120.0 | H | H | 1.88 | 0.00496 | OE |
| glyceraldehyde-3-phosphate dehydrogenase, testis-specific | 7657116 | 179.3 | 190.7 | H | H | 1.89 | 0.01018 | OE |
| outer dense fiber protein 2 isoform 3 | 310750406 | 213.3 | 175.0 | H | H | 2.01 | 0.01799 | OE |
| glutathione S-transferase P | 4504183 | 42.3 | 30.7 | M | M | 2.01 | 0.04972 | OE |
| lactotransferrin isoform 1 precursor | 54607120 | 992.3 | 842.0 | H | H | 2.03 | 0.00031 | OE |
| carbonic anhydrase 4 precursor | 4502519 | 20.3 | 18.3 | M | L | 2.09 | 0.00352 | OE |
| proteasome subunit alpha type-5 isoform 1 | 23110942 | 23.3 | 21.3 | M | M | 2.11 | 0.00785 | OE |
| laminin subunit beta-2 precursor | 119703755 | 74.0 | 59.7 | M | M | 2.13 | 0.00101 | OE |
| nuclear pore membrane glycoprotein 210-like isoform 1 precursor | 117414168 | 108.0 | 95.0 | H | H | 2.16 | 0.00703 | OE |
| sorbitol dehydrogenase | 156627571 | 101.7 | 72.7 | H | M | 2.17 | 0.00162 | OE |
| proteasome subunit alpha type-2 | 4506181 | 26.0 | 22.7 | M | M | 2.18 | 0.03439 | OE |
| glyceraldehyde-3-phosphate dehydrogenase | 7669492 | 111.7 | 98.7 | H | H | 2.22 | 0.00196 | OE |
| phosphatidylethanolamine-binding protein 1 preproprotein | 4505621 | 27.7 | 19.3 | M | M | 2.24 | 0.00071 | OE |
| phosphoglycerate kinase 1 | 4505763 | 21.0 | 19.7 | M | M | 2.26 | 0.00080 | OE |
| chloride intracellular channel protein 1 | 14251209 | 40.7 | 24.3 | M | M | 2.35 | 0.00620 | OE |
| dihydrolipoyllysine-residue succinyltransferase component of 2-oxoglutarate dehydrogenase complex, mitochondrial isoform 1 precursor | 19923748 | 20.0 | 18.7 | M | L | 2.41 | 0.00880 | OE |
| ras-related protein Rab-14 | 19923483 | 16.3 | 20.3 | M | M | 2.45 | 0.00356 | OE |
| glutathione S-transferase Mu 3 | 23065552 | 143.7 | 103.7 | H | H | 2.48 | 0.00018 | OE |
| very long-chain specific acyl-CoA dehydrogenase, mitochondrial isoform 1 precursor | 4557235 | 33.7 | 31.3 | M | M | 2.48 | 0.02284 | OE |
| lysosome-associated membrane glycoprotein 1 precursor | 112380628 | 27.7 | 19.3 | M | L | 2.49 | 0.00081 | OE |
| tektin-3 | 13994250 | 75.3 | 73.7 | M | M | 2.49 | 0.00074 | OE |
| glycerol kinase 2 | 41393575 | 16.7 | 16.7 | L | L | 2.51 | 0.00457 | OE |
| cysteine-rich secretory protein 1 isoform 1 precursor | 327315372 | 65.7 | 58.0 | M | M | 2.53 | 0.00081 | OE |
| L-lactate dehydrogenase C chain | 4504973 | 62.0 | 62.0 | M | M | 2.54 | 0.00031 | OE |
| olfactomedin-4 precursor | 32313593 | 54.0 | 38.0 | M | M | 2.54 | 0.00023 | OE |
| histone H2B type 1-A | 24586679 | 33.3 | 17.7 | M | L | 2.58 | 0.00454 | OE |
| NADH-cytochrome b5 reductase 2 | 47778923 | 13.3 | 9.7 | L | L | 2.62 | 0.00546 | OE |
| succinate dehydrogenase [ubiquinone] flavoprotein subunit, mitochondrial | 156416003 | 68.0 | 65.7 | M | M | 2.64 | 0.01617 | OE |
| proteasome subunit alpha type-7 | 4506189 | 22.3 | 16.0 | M | L | 2.71 | 0.00366 | OE |
| protein DJ-1 | 31543380 | 17.0 | 13.0 | L | L | 2.72 | 0.00931 | OE |
| carnitine O-palmitoyltransferase 2, mitochondrial precursor | 4503023 | 66.7 | 51.0 | M | M | 2.73 | 0.01296 | OE |
| angiotensin-converting enzyme isoform 1 precursor | 4503273 | 155.0 | 98.0 | H | H | 2.75 | 0.00686 | OE |
| malate dehydrogenase, mitochondrial precursor | 21735621 | 184.3 | 139.3 | H | H | 2.84 | 0.00262 | OE |
| hypoxanthine-guanine phosphoribosyltransferase | 4504483 | 19.3 | 13.0 | L | L | 2.86 | 0.00500 | OE |
| L-xylulose reductase isoform 1 | 7705925 | 25.3 | 17.0 | M | L | 3.02 | 0.01314 | OE |
| calmodulin | 58218968 | 32.7 | 20.3 | M | M | 3.02 | 0.02444 | OE |
| calreticulin precursor | 4757900 | 65.0 | 50.7 | M | M | 3.07 | 0.01199 | OE |
| dipeptidyl peptidase 4 | 18765694 | 129.3 | 76.0 | H | M | 3.09 | 0.00081 | OE |
| proteasome subunit beta type-3 | 22538465 | 23.0 | 14.3 | M | L | 3.11 | 0.02187 | OE |
| uromodulin precursor | 59850812 | 36.3 | 19.3 | M | L | 3.13 | 0.02547 | OE |
| peroxiredoxin-4 precursor | 5453549 | 26.3 | 16.3 | M | L | 3.17 | 0.01207 | OE |
| proteasome subunit alpha type-1 isoform 2 | 4506179 | 25.3 | 15.3 | M | L | 3.21 | 0.00700 | OE |
| proteasome subunit beta type-1 | 4506193 | 23.0 | 14.0 | M | L | 3.31 | 0.00106 | OE |
| ras GTPase-activating-like protein IQGAP1 | 4506787 | 37.0 | 13.3 | M | L | 3.32 | 0.00307 | OE |
| ATP-citrate synthase isoform 1 | 38569421 | 24.0 | 8.7 | M | L | 3.34 | 0.00487 | OE |
| epididymal secretory protein E1 precursor | 5453678 | 18.3 | 9.7 | L | L | 3.35 | 0.00345 | OE |
| testis-expressed protein 101 isoform 1 | 194018544 | 25.3 | 17.0 | M | L | 3.36 | 0.00486 | OE |
| poly(rC)-binding protein 1 | 222352151 | 20.7 | 10.7 | M | L | 3.36 | 0.03478 | OE |
| serine/threonine-protein phosphatase 2A catalytic subunit beta isoform | 57222565 | 12.7 | 8.0 | L | L | 3.55 | 0.00388 | OE |
| G-protein coupled receptor 64 isoform 6 precursor | 296317297 | 37.3 | 20.7 | M | M | 3.56 | 0.00060 | OE |
| proteasome subunit beta type-5 isoform 1 | 4506201 | 43.0 | 24.3 | M | M | 3.64 | 0.03641 | OE |
| WD repeat-containing protein 16 isoform b | 124028512 | 29.7 | 18.0 | M | L | 3.67 | 0.02011 | OE |
| fibronectin isoform 3 preproprotein | 16933542 | 1510.0 | 563.0 | H | H | 3.71 | 0.00021 | OE |
| protein-glutamine gamma-glutamyltransferase 4 | 156627577 | 155.3 | 33.7 | H | M | 3.72 | 0.00046 | OE |
| filamin-B isoform 1 | 256222411 | 47.7 | 18.7 | M | L | 3.79 | 0.00551 | OE |
| proteasome subunit alpha type-4 isoform 1 | 4506185 | 25.3 | 12.3 | M | L | 3.84 | 0.01806 | OE |
| ferritin, mitochondrial precursor | 29126241 | 43.7 | 22.0 | M | M | 3.85 | 0.00145 | OE |
| superoxide dismutase [Mn], mitochondrial isoform A precursor | 67782305 | 23.3 | 11.0 | M | L | 3.95 | 0.00238 | OE |
| laminin subunit alpha-5 precursor | 21264602 | 100.3 | 41.0 | H | M | 4.13 | 0.00219 | OE |
| proteasome subunit alpha type-6 | 23110944 | 40.0 | 18.0 | M | L | 4.32 | 0.01442 | OE |
| serpin B6 | 41152086 | 50.7 | 32.3 | M | M | 4.41 | 0.00001 | OE |
| glutathione reductase, mitochondrial isoform 1 precursor | 50301238 | 25.0 | 16.0 | M | L | 4.43 | 0.00070 | OE |
| laminin subunit gamma-1 precursor | 145309326 | 81.3 | 36.0 | H | M | 4.43 | 0.00020 | OE |
| alpha-mannosidase 2C1 | 46852164 | 33.3 | 14.7 | M | L | 4.55 | 0.00016 | OE |
| proteasome subunit alpha type-3 isoform 2 | 23110939 | 26.3 | 11.0 | M | L | 4.71 | 0.02399 | OE |
| brain acid soluble protein 1 | 30795231 | 43.0 | 19.7 | M | L | 4.98 | 0.00099 | OE |
| medium-chain specific acyl-CoA dehydrogenase, mitochondrial isoform b precursor | 187960098 | 42.0 | 22.0 | M | M | 5.02 | 0.00017 | OE |
| carnitine O-acetyltransferase precursor | 21618331 | 8.0 | 4.3 | L | VL | 5.05 | 0.22697 | OE |
| basigin isoform 2 precursor | 38372925 | 8.3 | 4.7 | L | VL | 5.22 | 0.00264 | OE |
| cytosol aminopeptidase | 41393561 | 115.3 | 59.3 | H | M | 5.28 | 0.00000 | OE |
| serum albumin preproprotein | 4502027 | 270.0 | 83.0 | H | H | 5.61 | 0.00232 | OE |
| aspartyl aminopeptidase | 156416028 | 20.3 | 9.3 | M | L | 6.15 | 0.00309 | OE |
| protein NipSnap homolog 3A | 22267436 | 23.3 | 7.0 | M | VL | 6.23 | 0.01272 | OE |
| 4-trimethylaminobutyraldehyde dehydrogenase | 115387104 | 45.3 | 17.7 | M | L | 6.38 | 0.00052 | OE |
| CD177 antigen precursor | 110735433 | 19.3 | 8.3 | L | L | 6.41 | 0.00155 | OE |
| malate dehydrogenase, cytoplasmic isoform 1 | 312283701 | 28.0 | 10.0 | M | L | 6.64 | 0.00040 | OE |
| galectin-3-binding protein precursor | 5031863 | 35.0 | 9.7 | M | L | 6.67 | 0.00404 | OE |
| purine nucleoside phosphorylase | 157168362 | 25.0 | 7.3 | M | VL | 6.68 | 0.01266 | OE |
| UPF0577 protein KIAA1324 precursor | 38569482 | 8.7 | 2.7 | L | VL | 6.69 | 0.00826 | OE |
| glutamate carboxypeptidase 2 isoform 1 | 4758398 | 50.3 | 12.7 | M | L | 6.75 | 0.00166 | OE |
| erythrocyte band 7 integral membrane protein isoform a | 38016911 | 31.3 | 11.3 | M | L | 7.12 | 0.01272 | OE |
| adenosylhomocysteinase isoform 1 | 9951915 | 36.3 | 13.0 | M | L | 7.21 | 0.00009 | OE |
| dihydrolipoyl dehydrogenase, mitochondrial precursor | 91199540 | 100.0 | 44.3 | H | M | 7.21 | 0.00019 | OE |
| growth arrest-specific protein 6 isoform 1 precursor | 4557617 | 11.3 | 1.3 | L | VL | 7.49 | 0.00689 | OE |
| gamma-glutamyltranspeptidase 1 precursor | 73915090 | 28.0 | 7.7 | M | VL | 7.84 | 0.00117 | OE |
| thioredoxin reductase 2, mitochondrial precursor | 22035672 | 20.0 | 6.3 | M | VL | 9.14 | 0.00321 | OE |
| D-dopachrome decarboxylase | 4503291 | 9.0 | 1.0 | L | VL | 9.23 | 0.00495 | OE |
| maltase-glucoamylase, intestinal | 221316699 | 114.3 | 22.7 | H | M | 9.81 | 0.00107 | OE |
| glucose-6-phosphate isomerase isoform 2 | 18201905 | 69.7 | 20.3 | M | M | 9.84 | 0.00080 | OE |
| aspartate aminotransferase, mitochondrial precursor | 73486658 | 76.0 | 15.0 | M | L | 12.65 | 0.00066 | OE |
| aldose reductase | 4502049 | 12.7 | 2.3 | L | VL | 13.63 | 0.00355 | OE |
| glucosamine-6-phosphate isomerase 1 | 13027378 | 10.3 | 1.3 | L | VL | 14.66 | 0.00402 | OE |
| ferritin heavy chain | 56682959 | 9.3 | 1.0 | L | VL | 16.68 | 0.00083 | OE |
| aspartate aminotransferase, cytoplasmic | 4504067 | 34.0 | 5.3 | M | VL | 16.98 | 0.00092 | OE |
| zinc-alpha-2-glycoprotein precursor | 4502337 | 54.0 | 8.3 | M | L | 17.21 | 0.00310 | OE |
| ectonucleotide pyrophosphatase/phosphodiesterase family member 3 | 111160296 | 20.7 | 2.3 | M | VL | 17.62 | 0.00861 | OE |
| apolipoprotein D precursor | 4502163 | 11.3 | 1.0 | L | VL | 22.98 | 0.00072 | OE |
| homogentisate 1,2-dioxygenase | 115527117 | 14.3 | 1.7 | L | VL | 25.62 | 0.00460 | OE |
| 60S ribosomal protein L5 | 14591909 | 9.3 | 0.7 | L | VL | 25.88 | 0.00146 | OE |
| kielin/chordin-like protein isoform 1 precursor | 209571519 | 4.7 | 0.0 | VL | ni | 2 only | 0.00007 | Unique to Group 2 |
| 5-oxoprolinase | 48314820 | 6.3 | 0.0 | VL | ni | 2 only | 0.00018 | Unique to Group 2 |
| xaa-Pro dipeptidase isoform 1 | 149589008 | 4.7 | 0.0 | VL | ni | 2 only | 0.00068 | Unique to Group 2 |
| serotransferrin precursor | 4557871 | 14.0 | 0.0 | L | ni | 2 only | 0.00005 | Unique to Group 2 |
| basement membrane-specific heparan sulfate proteoglycan core protein precursor | 126012571 | 13.7 | 0.0 | L | ni | 2 only | 0.00078 | Unique to Group 2 |
| cysteine-rich secretory protein 2 precursor | 215490018 | 12.0 | 0.0 | L | ni | 2 only | 0.00078 | Unique to Group 2 |
| amiloride-sensitive amine oxidase [copper-containing] precursor | 73486661 | 11.7 | 0.0 | L | ni | 2 only | 0.00199 | Unique to Group 2 |
| glycerol-3-phosphate dehydrogenase 1-like protein | 24307999 | 8.7 | 0.0 | L | ni | 2 only | 0.00230 | Unique to Group2 |
| N(G),N(G)-dimethylarginine dimethylaminohydrolase 1 isoform 1 | 6912328 | 9.0 | 0.0 | L | ni | 2 only | 0.00686 | Unique to Group 2 |

^*^ni: not identified; H:high abundance; M: medium abundance; L:low abundance; VL: very low abundance.
