# Supplementary material for: Mitochondrial dysfunction signatures in idiopathic primary male infertility: a validated proteomics-based diagnostic approach
Source: Front Reprod Health. 2024 Dec 12;6:1479568. doi: 10.3389/frph.2024.1479568 (PMC11669654; doi:10.3389/frph.2024.1479568)
Supplement: Supplementary file 3 [file Table3.docx]

**Figures legends**

Figure 1. Study flowchart.

Figure 2. STRING analysis of protein-protein interactions. Network Status: number of nodes, 141; number of edges, 711; average node degree, 10.1; expected number of edges, 290; average local clustering coefficient, 0.433, and protein-protein interaction(PPI) enrichment *P*-value < 1.0E-16. ^a^Count in network refers to the number of DEP’ included in the STRING interaction network.

Figure 3. Western blot validation of three differentially expressed proteins in patients with idiopathic primary infertility (*n*=4) and in healthy fertile donors (*n*=4). (A) Representative image of WB , (B) Quantification of the WB results. Results are expressed as the mean fold change ± standard error of the mean and normalised to that of the control protein.PRDX5 and SOD were significantly decreased in patient sperm samples when compared with that of healthy donors. GAPDH. GSR, glutathione disulphide reductase; SOD, superoxide dismutase; PRDX5, peroxiredoxin-5; GAPDH, glyceraldehyde 3-phosphate dehydrogenase.
